# Structural alterations of the salience network in patients with insular glioma

**DOI:** 10.1002/brb3.2969

**Published:** 2023-03-28

**Authors:** Guanjie Hu, Zhiqiang Wu, Bowen Cao, Qinyu Shi, Zifeng Zhang, Xiao Fan, Yao Tang, Zhangchun Cheng, Xiefeng Wang, Shenqi Jing, Tao Li, Junxia Zhang, Yongping You

**Affiliations:** ^1^ Department of Neurosurgery The First Affiliated Hospital of Nanjing Medical University Nanjing China; ^2^ Department of Medical Informatics, School of Biomedical Engineering and Informatics Nanjing Medical University Nanjing China; ^3^ Center for Data Management The First Affiliated Hospital of Nanjing Medical University (Jiangsu Province Hospital) Nanjing China; ^4^ Institute of Medical Informatics and Management Nanjing Medical University Nanjing China; ^5^ Shanghai Synyi Medical Technology Co., Ltd Shanghai China; ^6^ Institute for Brain Tumors, Jiangsu Collaborative Innovation Center for Cancer Personalized Medicine Nanjing Medical University Nanjing China

**Keywords:** fractional anisotropy, gray matter volume, insula glioma, topological network

## Abstract

**Objective:**

The structural alteration that occurs within the salience network (SN) in patients with insular glioma is unclear. Therefore, we aimed to investigate the changes in the topological network and brain structure alterations within the SN in patients with insular glioma.

**Methods:**

We enrolled 46 patients with left insular glioma, 39 patients with right insular glioma, and 21 demographically matched healthy controls (HCs). We compared the topological network, gray matter (GM) volume, and fractional anisotropy (FA) between HCs and patients after controlling for the effects of age and gender.

**Results:**

Patients with insular glioma showed topological network decline mainly in the insula, basal ganglia region, and anterior cingulate cortex (ACC). Compared with HCs, patients primarily showed GM volume increased in the ACC, inferior temporal gyrus (ITG), superior temporal gyrus (STG), temporal pole: middle temporal gyrus (TPOmid), insula, middle temporal gyrus (MTG), middle frontal gyrus, and superior occipital gyrus (SOG), but decreased in TPOmid, ITG, temporal pole: superior temporal gyrus, and SOG. FA declined mainly in the STG, MTG, ACC, superior frontal gyrus, and SOG, and also showed an increased cluster in SOG.

**Conclusions:**

FA represents the integrity of the white matter. In patients with insular glioma, decreased FA may lead to the destruction of the topological network within the SN, which in turn may lead to the decrease of network efficiency and brain function, and the increase of GM volume may compensate for these changes. Overall, this pattern of structural changes provides new insight into the compensation model of insular glioma.

## Graphical abstract

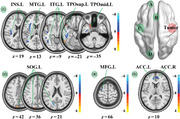

## 1 INTRODUCTION

Glioma is the most common tumor operated on in neurosurgery. However, even when well‐treated, less than 5% of patients with glioma survive for 5 years (Duffau & Taillandier, 2015; Ghinda et al., 2018; Gusyatiner & Hegi, 2018). Gliomas in the core of the insula result in several functional declines in patients, such as deficits in language, body movement, and cognitive function, which are major challenges in diagnosis and treatment (Liu et al., 2021).

The brain contains a variety of functional networks that serve unique functions. Power et al. (2011) segmented brain networks from resting‐state functional magnetic resonance imaging (fMRI) based on the functional boundary method and obtained 10 different brain networks. However, Yeo et al. (2011) provide seven and 17 networks based on rough segmentation and fine segmentation, respectively, using resting‐state fMRI data of 1000 healthy individuals. Although the functional networks are defined slightly differently in the literature, the default mode network (DMN), executive control network (ECN), and salience network (SN) are recognized as three of the most important functional brain networks (Ji et al., 2019). The SN, as one of the advanced neural networks, plays a crucial role in processing complex tasks in the human brain. The SN contains the anterior insula (AI) and anterior cingulate cortex (ACC) (Chong et al., 2017; Ku et al., 2020). The main role of the SN is to detect, filter, and highlight stimulation, then recruit part of the relevant functional network to detect and integrate emotional and sensory stimulation, as well as in switching between the DMN and ECN (Song et al., 2021).

The SN has characteristic changes in a variety of brain diseases, and some characteristic changes play the role of biomarkers in disease progression. Song et al. (2021) found specific functional alternations within the SN and interactions between the SN and other networks in mild cognitive impairment (MCI), which may provide new insights into predicting the progression of MCI and new targets for appropriate interventions to delay further cognitive decline by meta‐analysis. Longitudinal analysis of fMRI showed that the functional connectivity (FC) within the SN decreased after repetitive transcranial magnetic stimulation (rTMS), but the FC between the SN and the posterior DMN (pDMN) increased, and the regional activity of both the SN and pDMN increased in patients with depression, which provided evidence for the treatment of depression with rTMS (Godfrey et al., 2022). Migraineurs with aura exhibited more fluctuating connections in the SN (Vereb et al., 2020), schizophrenia patients showed abnormalities in FC between SN and ECN regions (Smucny et al., 2017), and the molecular level also showed that D2 receptor levels in the SN and the right parahippocampal may contribute to memory impairment in patients with Parkinson disease (Christopher et al., 2015). Glioma also caused some regional or distal changes within or across the SN due to the infiltration growth of glioma (Sparacia et al., 2020; Yang, Gohel, et al., 2021). However, the effect of insular gliomas on the SN is still unclear. Insular gliomas involve constituent brain areas of the SN, and in fact, studies have shown that the insular is functionally complex and participates in sensorimotor, olfactory, social–emotional, and cognitive functions (Kurth et al., 2010; Uddin et al., 2017). Diener et al. (2012) also emphasized the key role of the insular in altered emotion and cognition. In glioma patients, lesions in the insular may cause language impairment in some patients after surgery, and vocabulary ability, verbal memory, and learning ability may also be affected by insular lesions (Mandonnet, 2019; Wu et al., 2011; Zarino et al., 2021). Therefore, investigation of structural brain alterations within the SN may provide a deeper understanding of the neurological changes that occur in patients with insular glioma.

Along with functional networks, topological networks provide an important tool to represent brain structure. Topological networks are constructed based on fiber tracking results of diffusion tensor image (DTI). The white matter (WM) fiber bundles of each subject can be obtained by fiber tracking, and the weighted structural network of each subject can be obtained by considering the brain areas as the nodes of the topological network and the fiber connections as the weights between nodes and edges of the topological network (Basser et al., 2000; Cunningham et al., 2017). DTI is a magnetic resonance imaging (MRI) method with high sensitivity in detecting microstructural alterations of WM. Topological networks constructed by DTI have attracted extensive attention in neuroscience research, and they reflect the topological changes and connectivity of structural brain networks (Bassett et al., 2017). Many studies have explored WM changes using DTI (Bosch et al., 2012; Mayo et al., 2018). In fact, DTI has also been used to assess structural changes in low‐grade gliomas after chemotherapy (Castellano et al., 2016). Fractional anisotropy (FA) of DTI characterizes the constraint degree of water diffusion in a certain direction, which is sensitive to the alignment of WM fibers and the structural integrity of myelination (Li et al., 2015; Melhem et al., 2002). Changes in FA may imply changes in brain cognitive function (Raghavan et al., 2020). However, the human brain is a complex and interconnected network that maintains a balance between regional isolation and functional integration (Zhou et al., 2021). Therefore, DTI can be used to better understand the topological alterations of WM in gliomas.

Gray matter (GM) is also an important marker of brain structure. Many studies have confirmed that cognitive decline and epilepsy occurrence is related to alterations of brain structure and functional networks in patients with insular glioma (Almairac et al., 2021; Wu et al., 2011). Almairac et al. (2018) used voxel‐based morphological analysis to uncover compensatory GM increases in the contralateral insula in patients with unilateral insular glioma. This finding has been confirmed in the work of other researchers. Hu, Hu, et al. (2020) found similar changes in GM volume in temporal glioma, which was highly correlated with cognitive functions such as visuospatial mapping. Huang et al. (2021) further demonstrated GM compensation in insular glioma. However, Yuan et al. (2020) found the opposite results, with contralateral homotopic decompensation of GM volume in insular glioma patients. Thus, the pattern of GM changes within the SN in patients with insular glioma needs further investigation and verification.

Topological networks constructed by DTI could illustrate changes to brain structure in patients with insular glioma. Insular gliomas involve constituent brain areas of the SN, but the brain structure alterations within the SN remain unclear in patients with insular glioma. Therefore, we investigated the changes in the topological network and structural brain alterations within the SN in patients with insular glioma. We hypothesized that the topological network, GM, and FA of WM may reveal special change patterns within the SN in patients with insular glioma.

## 2 METHODS

### 2.1 Patient enrollment

We collected MRI data of patients with insular glioma from the First Affiliated Hospital of Nanjing Medical University (from 2015 to 2021). All patients underwent MRI examinations and had confirmed insular glioma with postoperative pathology. Originally, 98 patients with pathologically confirmed insular glioma were included; 51 patients had lesions located in the left hemisphere (left insular glioma [LIG]), and 47 patients had lesions located in the right hemisphere (right insular glioma [RIG]). The glioma cores in the patients in this study were all located in the insula and confined to one hemisphere. Exclusion criteria were as follows: (1) drug or alcohol abuse or dependence; (2) head trauma; (3) MRI contraindications; (4) other brain diseases; (5) brain midline deviation; and (6) poor data quality and MRI image preprocessing failure. We also enrolled 21 healthy controls (HCs) demographically matched on age and gender without any brain disorders, head trauma, or psychological disorders. This study was approved by the Institutional Ethical Committee for Clinical Research of the First Affiliated Hospital of Nanjing Medical University.

### 2.2 MRI data acquisition

Three‐dimensional (3D) T1‐weighted MRI images and DTI images of each subject were acquired from a 3.0 Tesla MRI scanner (Verio, Siemens) in the Department of Radiology at the First Affiliated Hospital of Nanjing Medical University from 2015 to 2021. Sequence parameters for 3D T1‐weighted MRI images were as follow: repetition time (TR) = 1600 ms; echo time (TE) = 2.48 ms; inversion time (TE) = 900 ms; matrix = 256 × 256; flip angle = 9; thickness = 1.5 mm; echo train length = 1; pixel bandwidth = 170. The parameters for DTI were as follows: TR = 4900 ms; TE = 95 ms; matrix = 128 × 128; flip angle = 90; thickness = 3 mm; echo train length = 50; pixel bandwidth = 1500; spacing between slices = 3 mm; 26 (patients) or 62 (HCs) noncollinear directions of diffusion encoding (*b* = 1000 s/mm^2^ for each direction). We used different parameters to optimize and improve the imaging protocol, which were independent of the purpose of the study. The data will not be made public due to privacy protection, but it can be provided by the author upon reasonable request from the readers.

### 2.3 Image preprocessing

All images were reviewed by a neuroradiologist to exclude images with the head moving or midline offset. Three‐dimensional T1‐weighted images were preprocessed with the Diffeomorphic Anatomical Registration Through Exponentiated Lie Algebra (DARTEL) algorithm using the Data Processing and Analysis of Brain Imaging (DPABI) with the following preprocessing steps (Ashburner & Friston, 2009; Yan et al., 2016): selection of the anterior commissure as the origin (coordinates 0, 0, 0); dividing each image into GM, WM, and cerebrospinal fluid; smoothing the images and resampling to MNI (Montreal Neurological Institute) space (1.5 × 1.5 × 1.5 mm^3^) with 8‐mm full width at half‐maximum (FWHM) Gaussian kernel. The detailed preprocessing steps of 3D T1‐weighted images are described in Supporting Information S1.

The DTI images were preprocessed using the Pipeline for Analyzing braiN Diffusion imAges (PANDA) toolbox based on FMRIB Software Library V5.0 (Cui et al., 2013; Jenkinson et al., 2012). The preprocessing steps were as follows: DICOM format conversion to NIFTI format; skull removement; head motion artifact and eddy current correction; calculation of the diffusion matrix (the correlation between the dispersion rate sequence of a voxel and that of an adjacent voxel); normalization; smoothing with FWHM of 6 mm; deterministic fiber tracking. In the process of fiber tracking, we set the propagation algorithm of Fiber Assignment by Continuous Tracking (FACT), FA threshold of 0.2–1.0, and angle threshold of 45° (Basser et al., 2000; Feng et al., 2021). Segmentation of the brain using GM mapping resulted in 90 regions of interest (ROIs) using the Anatomical Automatic Labeling (AAL) atlas. Each of these regions acts as nodes in the network, while WM fibers that were tracked formed the edges in the network (Hu, Qian, et al., 2020; Li et al., 2021).

### 2.4 Statistical analysis

#### 2.4.1 Demographics characteristics

We performed one‐way ANOVA to compare age between the groups (HCs, patients with LIG, and patients with RIG). The sex ratio and tumor grade were determined by the chi‐square test. Tumor size was analyzed by the two‐sample *t*‐test in LTG and RTG. The alpha level for significance was .05. We performed these statistical analyses using the Statistical Package for the Social Sciences (SPSS) for Windows, version 22 (SPSS, Chicago, IL, USA).

#### 2.4.2 Comparison of the topological network

The brain network constructed by using the average FA value between each region of the brain atlas is referred to as the FA network (Li et al., 2021). We compared the FA network between HCs and patients with LIG with age and sex as covariates using the Gretna toolbox (Wang et al., 2015). Similarly, we performed the statistical analysis for the patients with RIG. The brain network constructed from the number of fibers between each region of the brain map is referred to as the FN network (Li et al., 2021). We conducted the same statistical analyses for both the FA network and the FN network. We performed false discovery rate (FDR) correction in network analysis and set the alpha level to .05.

#### 2.4.3 Voxel‐wise statistical analysis

For the GM volume, we performed a two‐sample *t*‐test between HCs and patients within the smoothed GM images using Data Processing & Analysis for Brain Imaging (DPABI) toolbox (Yan et al., 2016). The SN consists of AI and ACC (Chong et al., 2017; Ku et al., 2020), and the tumor core of the patient involved the insula in our study. Therefore, according to the regions of the SN, we first created three ROIs as masks using the WFU‐Pickatlas toolbox, including the ACC, left temporal insula lobe, and right temporal insula lobe. The DMN and ECN are closely related to the SN, and both involve the frontal lobe region (Song et al., 2021). Therefore, we also created the left and right frontal lobes as masks. Furthermore, to investigate voxel changes in other brain regions, left and right occipital lobe masks were created for statistical analysis. We compared the GM volume between HCs and patients with LIG within the ACC, right temporal insula lobe, right frontal lobe, and right occipital lobe masks. Similarly, we compared the GM volume between HCs and patients with RIG within the ACC, left temporal insula lobe, left frontal lobe, and left occipital lobe masks. To avoid the influence of the tumor itself, we did not perform statistical analysis on the tumor side. We performed FDR correction in GM analysis and set the alpha level to .05 with the cluster size >30 voxels.

Regarding the FA, we also performed a two‐sample *t*‐test at the voxel level in the smoothed FA images between HCs and patients using DPABI (Yan et al., 2016). We compared the FA images between HCs and patients with LIG within the ACC, right temporal insula lobe, right frontal lobe, and right occipital lobe masks. We compared the FA images between HCs and patients with RIG within the ACC, left temporal insula lobe, left frontal lobe, and left occipital lobe masks. We performed threshold‐free cluster extraction with family‐wise error correction in the FA analysis and set the alpha level to .05 with the cluster size >30 voxels. Notably, the above statistical analyses at the voxel level were performed after correcting the confounding factors including age and sex.

## 3 RESULTS

### 3.1 Demographic characteristics

There were 85 patients with insular glioma that were included in this study, including 46 patients with LIG (mean age = 50.74 ± 12.47) and 39 patients with RIG (mean age = 50.97 ± 9.42). There was no significant difference in age (*p* = .215) and sex ratio (*p* = .464) among patients with LIG, patients with RIG, and HCs (mean age = 55.24 ± 4.33). Tumor grade (*p* = .890) and tumor size (*p* = .823) also existed no significant difference in LTG (tumor size = 41.64 ± 30.80) and RTG (tumor size = 43.14 ± 30.68). All subjects were right‐handed and of Chinese descent. The demographics of the subjects are listed in Table 1.

**TABLE 1:** Demographic characteristics of patients with gliomas and healthy controls

		Patients			
Variable		LIGs	RIGs	HCs	*p*
*N*		46	39	21	NA
Age, year		50.74 ± 12.47 (14–78)	50.97 ± 9.42 (24–75)	55.24 ± 4.33(42–59)	.215
Sex ratio, F/M, *n*		20/26	16/23	12/9	.464
Handedness		R	R	R	NA
Grade	Low	10	8	NA	.890
	High	36	31		
Tumor size, cm^3^		41.64 ± 30.80 (1.90–136.80)	43.14 ± 30.68 (8.80–138.60)	NA	.823

Abbreviations: F, female; HCs, healthy controls; LIGs, left insula gliomas; M, male; NA, not applicable; R, right; RIGs, right insula gliomas.

### 3.2 Topological network comparison between HCs and patients

**FIGURE 1:**
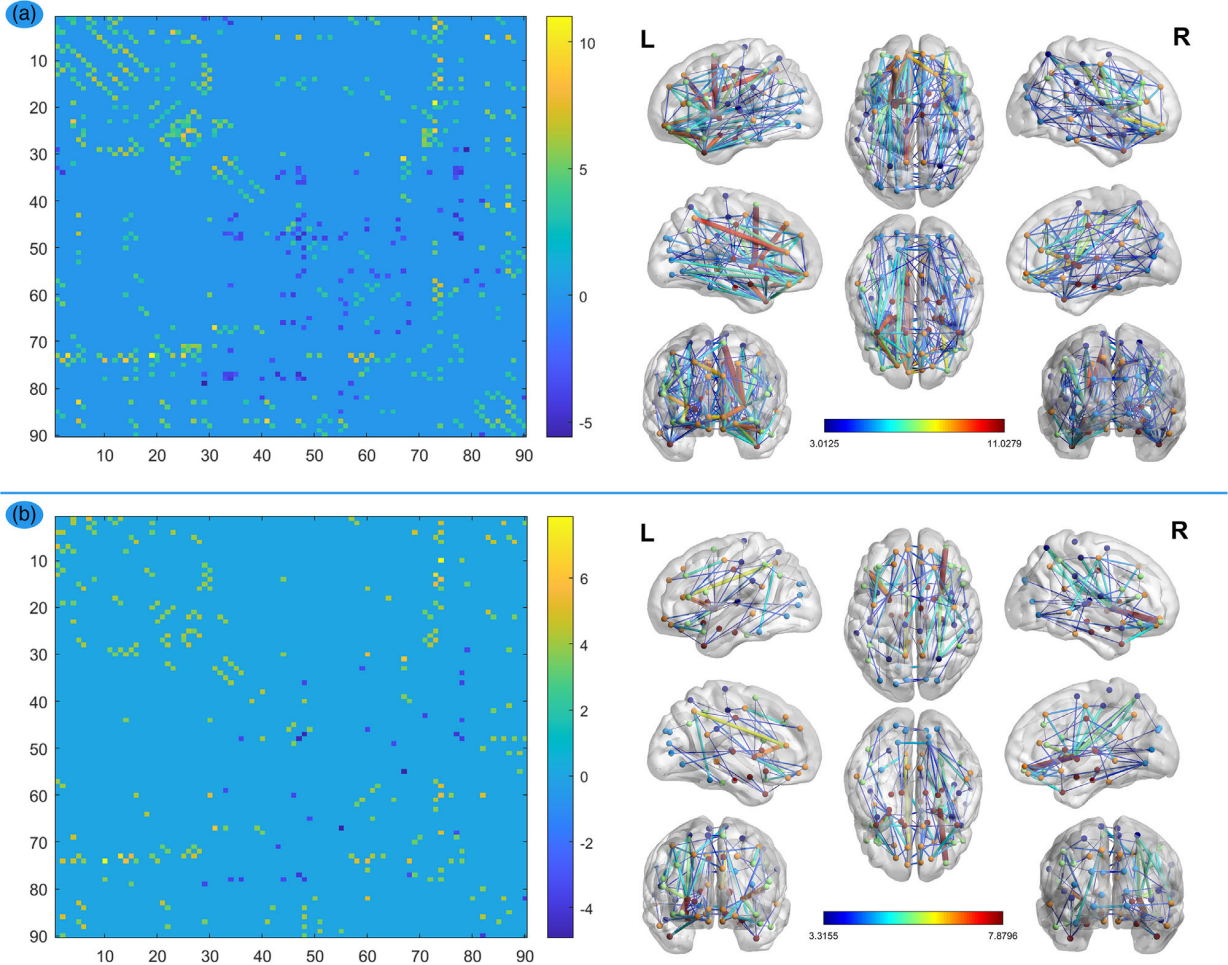
FA network comparisons between HCs and patients. The left column: the dots on these coordinates represent 90 brain regions in the automated anatomical labeling (AAL) template. The dots in different locations in the matrix represent the structural connectivity between the two different brain regions identified by the horizontal axis and vertical axis. The colors of the dots represent structural connection strength, quantified by the values on the right of the color bar. The warm dot color with a positive value represents decreased structural connection between the two brain regions in the patient group, compared with the HCs group, while the cool dot color with a negative value represents increased structural connection. The right column: it represented the significant connections (edges) between brain regions (nodes) visualized by BrainNet Viewer (NKLCNL, Beijing Normal University), which is the visualization of the matrix of the left column (*p* < .05, FDR‐corrected). LIGs, left insula gliomas; RIGs, right insula gliomas. (A) FA network comparisons between HCs and LIGs. FA network decline mainly located in the insula, basal ganglia region, and anterior cingulate cortex (ACC) in LIGs group compared with HCs group (*p* < .05, FDR‐corrected). (B) FA network comparisons between HCs and RIGs. FA network decline mainly located in the insula, basal ganglia region, and ACC in RIGs group compared with HCs group (*p* < .05, FDR‐corrected).

**FIGURE 2:**
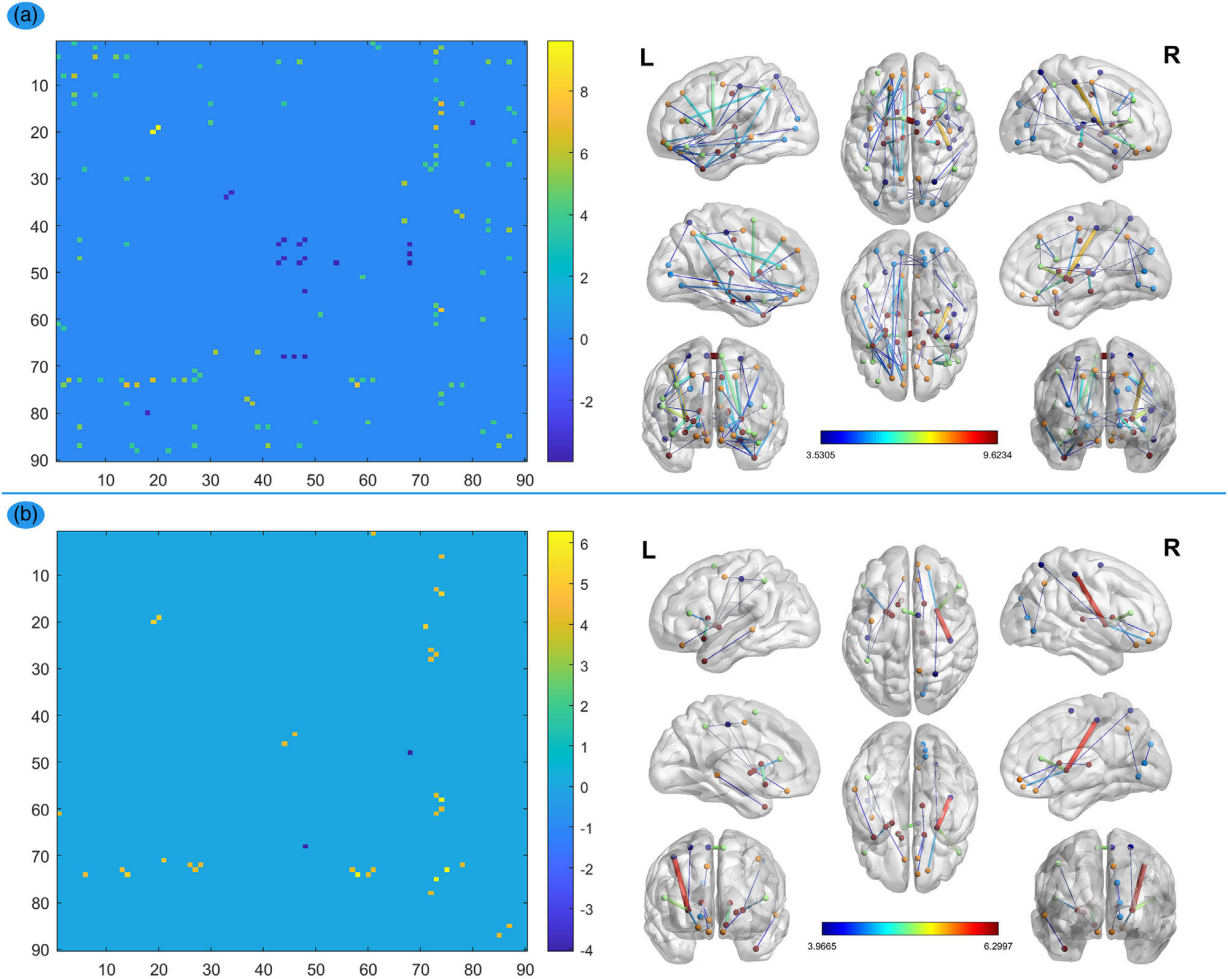
FN network comparisons between HCs and patients. The left column: the dots on these coordinates represent 90 brain regions in the automated anatomical labeling (AAL) template. The dots in different locations in the matrix represent the structural connectivity between the two different brain regions identified by the horizontal axis and vertical axis. The colors of the dots represent structural connection strength, quantified by the values on the right of the color bar. The warm dot color with a positive value represents decreased structural connection between the two brain regions in the patient group, compared with the HCs group, while the cool dot color with a negative value represents increased structural connection. The right column: it represented the significant connections (edges) between brain regions (nodes) visualized by BrainNet Viewer (NKLCNL, Beijing Normal University), which is the visualization of the matrix of the left column (*p* < .05, FDR‐corrected). LIGs, left insula gliomas; RIGs, right insula gliomas. (A) FN network comparisons between HCs and LIGs. FN network decline mainly located in the insula, basal ganglia region, and anterior cingulate cortex (ACC) in LIGs group compared with HCs group (*p* < .05, FDR‐corrected). (B) FN network comparisons between HCs and RIGs. FN network decline mainly located in the basal ganglia region in RIGs group compared with HCs group (*p* < .05, FDR‐corrected).

After FDR correction of the *p*‐value of <.05, we observed FA network decline mainly in the insula, basal ganglia region, and ACC in patients with LIG compared with HCs (Figure 1A). Similarly, compared with HCs, the patients with RIG showed FA network decline mainly in the insula, basal ganglia region, and ACC (Figure 1B). Regarding the FN network, the patients with LIG showed FN network decline mainly in the insula, basal ganglia region, and ACC (Figure 2A), but patients with RIG showed FN network decline mainly in the basal ganglia regions (Figure 2B).

### 3.3 GM volume comparison between HCs and patients

**FIGURE 3:**
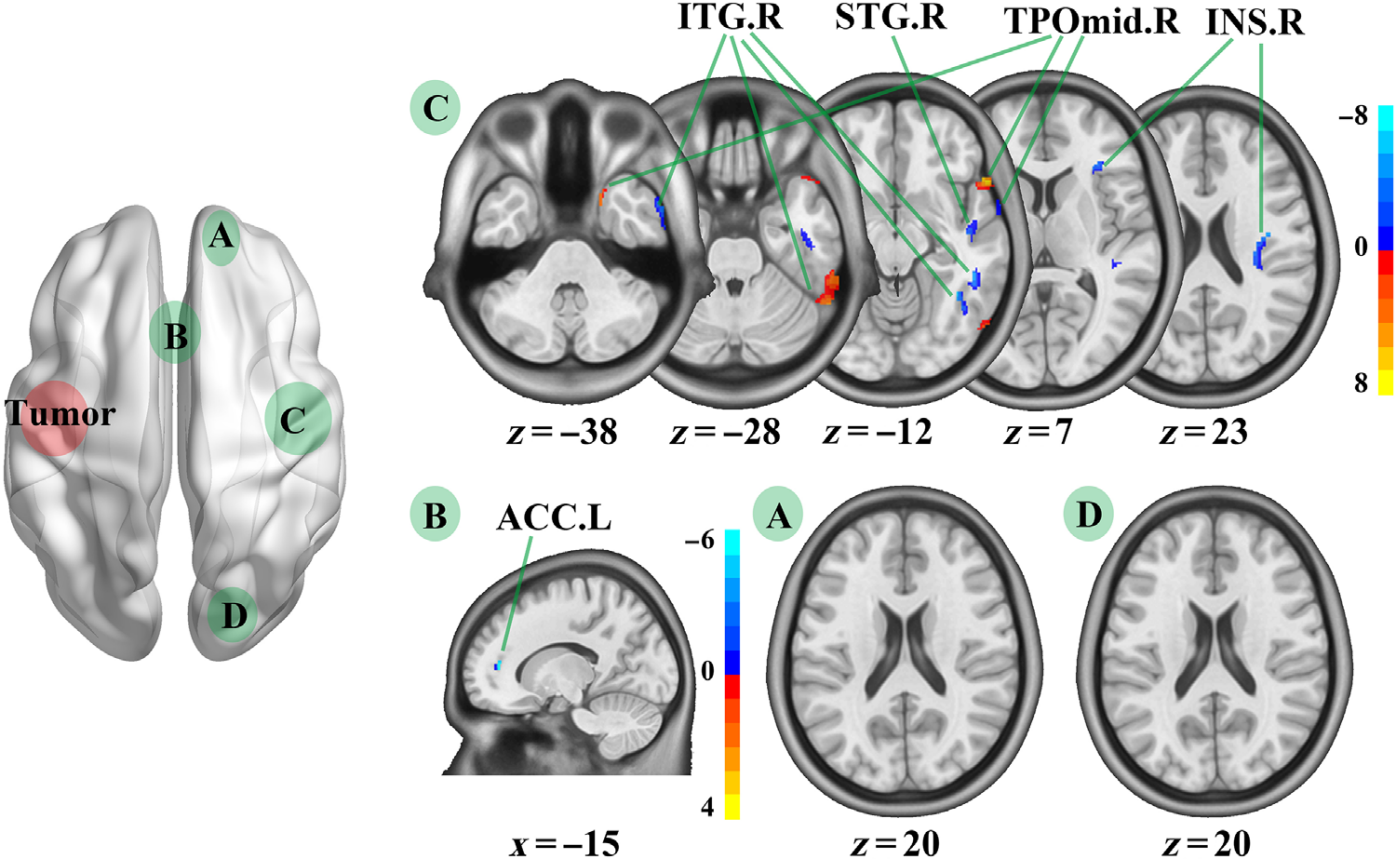
GM volume comparison between HCs and patients with left insula glioma. ACC.L, left anterior cingulate; ITG.R, right inferior temporal gyrus; STG.R, right superior temporal gyrus; TPOmid.R, temporal pole: middle temporal gyrus; INS.R, right insula; LIGs, left insula gliomas; RFG, right front gyrus; RTI, right temporal insula; ACC, anterior cingulate; ROG, right occipital gyrus. We compared GM volume between HCs and patients with left temporal insula glioma in RFG (A), ACC (B), RTI (C), and ROG (D). Compared with HCs, LIGs group showed increased GM volume in ACC.L, ITG.R, STG.R, TPOmid.R, and INS.R, but decreased GM volume in TPOmid.R and ITG.R.

**FIGURE 4:**
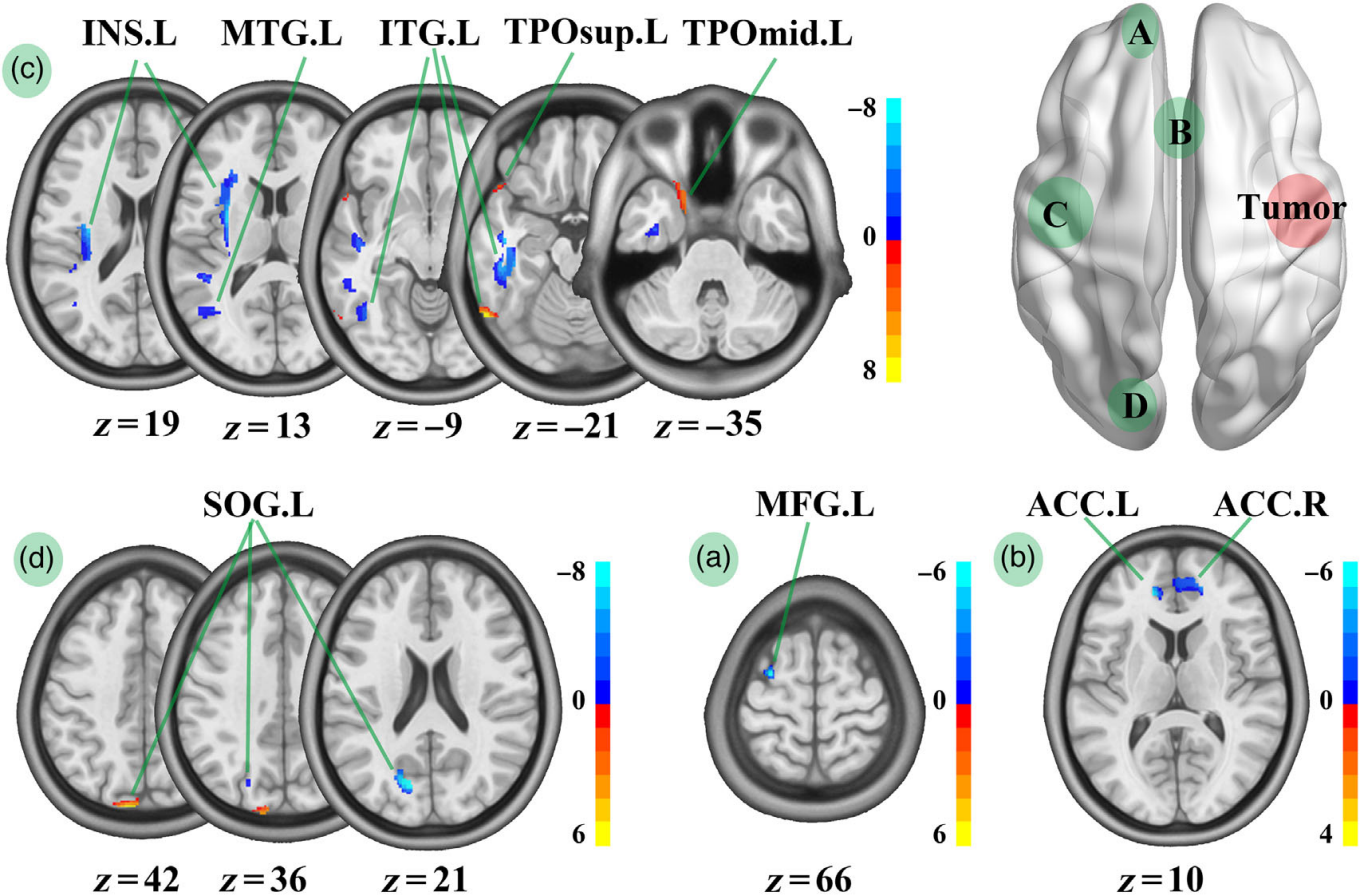
GM volume comparison between HCs and patients with right insula glioma. ACC.L, left anterior cingulate; ITG.L, left inferior temporal gyrus; MTG.L, left middle temporal gyrus; INS.L, left insula; ACC.R, right anterior cingulate cortex; MFG.L, left middle frontal gyrus; SOG.L, left superior occipital gyrus; TPOmid.L, left temporal pole: middle temporal gyrus; TPOsup.L, left temporal pole: superior temporal gyrus; RIGs, right insula gliomas; LFG, left front gyrus; LOG, left occipital gyrus; LTI, left temporal insula; ACC, anterior cingulate. We compared GM volume between HCs and patients with right temporal insula glioma in LFG (A), ACC (B), LTI (C), and LOG (D). Compared with HCs, RIGs group showed increased GM volume in ITG.L, MTG.L, INS.L, ACC.L, ACC.R, MFG.L, and SOG.L, but decreased in TPOmid.L, ITG.L, TPOsup.L, and SOG.L.

Through the voxel‐wise GM analysis, compared with HCs, patients with LIG showed increased GM volume in the left anterior cingulate cortex (ACC.L), right inferior temporal gyrus (ITG.R), right superior temporal gyrus (STG.R), right temporal pole: middle temporal gyrus (TPOmid.R), and right insula, but decreased GM volume in TPOmid.R and ITG.R (Table 2; Figure 3). Meanwhile, compared with HCs, patients with RIG showed increased GM volume in the left inferior temporal gyrus (ITG.L), left middle temporal gyrus, left insula, ACC.L, right anterior cingulate cortex, left middle frontal gyrus (MFG.L), and left superior occipital gyrus (SOG.L), but decreased GM volume in the left temporal pole: middle temporal gyrus, ITG.L, left temporal pole: superior temporal gyrus, and SOG.L (Table 2; Figure 4).

**TABLE 2:** Peak locations of regions: Comparison of GM between healthy controls and patients with glioma

	Areas	Peak location (MNI coordinates)			Cluster size (voxels)	*T*
		*X*	*Y*	*Z*		
LIGs	RTI mask					
	ITG.R	54	7.5	−45	308	−7.2341
	ITG.R	49.5	−36	−15	549	−6.8863
	ITG.R	39	−49.5	−12	124	−7.3444
	STG.R	45	−12	−15	511	−6.9532
	TPOmid.R	64.5	9	−15	76	−5.0197
	INS.R	31.5	30	7.5	41	−5.8938
	INS.R	37.5	6	16.5	174	−8.5185
	TPOmid.R	19.5	13.5	−37.5	42	5.8566
	TPOmid.R	57	21	−13.5	408	8.0059
	ITG.R	60	−60	−22.5	397	8.2868
	ACC mask					
	ACC.L	−15	42	12	30	−6.0316
RIGs	LTI mask					
	TPOmid.L	−16.5	7.5	−40.5	374	7.3752
	ITG.L	−42	−34.5	−13.5	2336	−9.8206
	ITG.L	−60	−63	−21	246	8.4127
	TPOsup.L	−54	19.5	−13.5	127	5.9503
	ITG.L	−42	−61.5	−9	128	−5.6671
	MTG.L	−48	−51	1.5	91	−4.7846
	INS.L	−31.5	−16.5	22.5	943	−9.1916
	INS.L	−31.5	−19.5	3	34	−7.1266
	MTG.L	−40.5	−61.5	10.5	202	−4.8767
	ACC mask					
	ACC.L	−15	43.5	10.5	244	−6.9308
	ACC.R	16.5	48	13.5	751	−6.6605
	ACC.L	−13.5	31.5	28.5	42	−5.4585
	LFG mask					
	MFG.L	−30	−1.5	66	44	−6.0275
	LOG mask					
	SOG.L	−13.5	85.5	4.5	40	−5.6865
	SOG.L	−13.5	−73.5	21	321	−8.127
	SOG.L	−9	−90	42	202	7.0266

*Note*: Compared with HCs, LIGs group showed increased GM volume in ACC.L, ITG.R, STG.R, TPOmid.R, and INS.R, but decreased GM volume in TPOmid.R and ITG.R. Compared with HCs, RIGs group showed increased GM volume in ITG.L, MTG.L, INS.L, ACC.L, ACC.R, MFG.L, and SOG.L, but decreased GM volume in TPOmid.L, ITG.L, TPOsup.L, and SOG.L.

Abbreviations: ACC, anterior cingulate; ACC.L, left anterior cingulate; ACC.R, right anterior cingulate cortex; GM, gray matter; INS.L, left insula; INS.R, right insula; ITG.L, left inferior temporal gyrus; ITG.R, right inferior temporal gyrus; LFG, left front gyrus; LIGs, left insula gliomas; LOG, left occipital gyrus; LTI, left temporal insula; MFG.L, left middle frontal gyrus; MNI, Montreal Neurological Institute; MTG.L, left middle temporal gyrus; RIGs, right insula gliomas; RTI, right temporal insula; SOG.L, left superior occipital gyrus; STG.R, right superior temporal gyrus; TPOmid.L, left temporal pole: middle temporal gyrus; TPOmid.R, temporal pole: middle temporal gyrus; TPOsup.L, left temporal pole: superior temporal gyrus.

### 3.4 FA map comparison between HCs and patients

**FIGURE 5:**
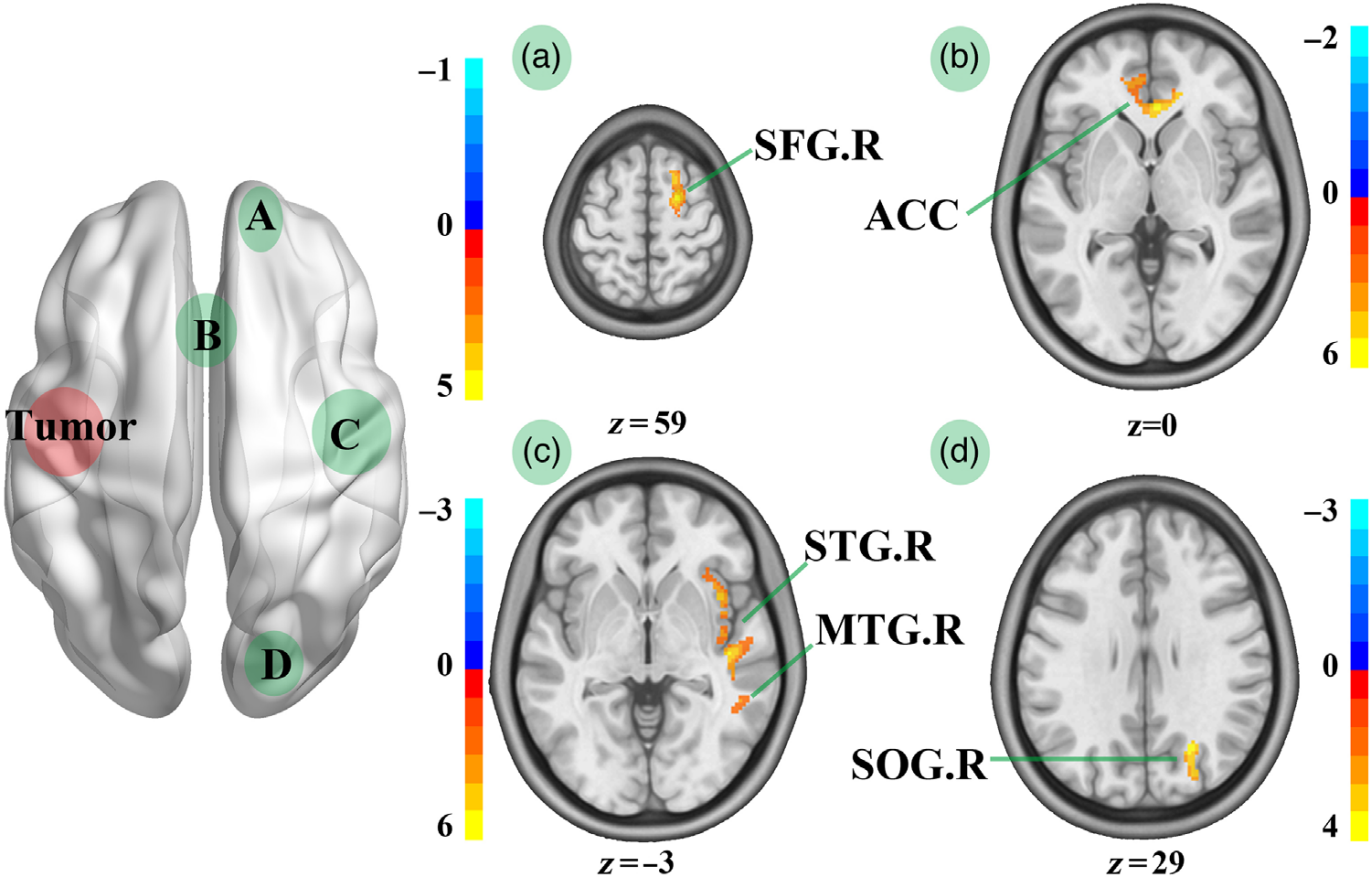
FA map comparison between HCs and patients with left insula glioma. STG.R, right superior temporal gyrus; MTG.R, right middle temporal gyrus; ACC, anterior cingulate; SFG.R, right superior frontal gyrus; SOG.R, right superior occipital gyrus; LIGs, left insula gliomas; RFG, right front gyrus; RTI, right temporal insula; ACC, anterior cingulate; ROG, right occipital gyrus. We compared FA between HCs and patients with left temporal insula glioma in RFG (A), ACC (B), RTI (C), and ROG (D). Compared with HCs, LIGs group showed decreased FA in STG.R, MTG.R, ACC, SFG.R, and SOG.R.

**FIGURE 6:**
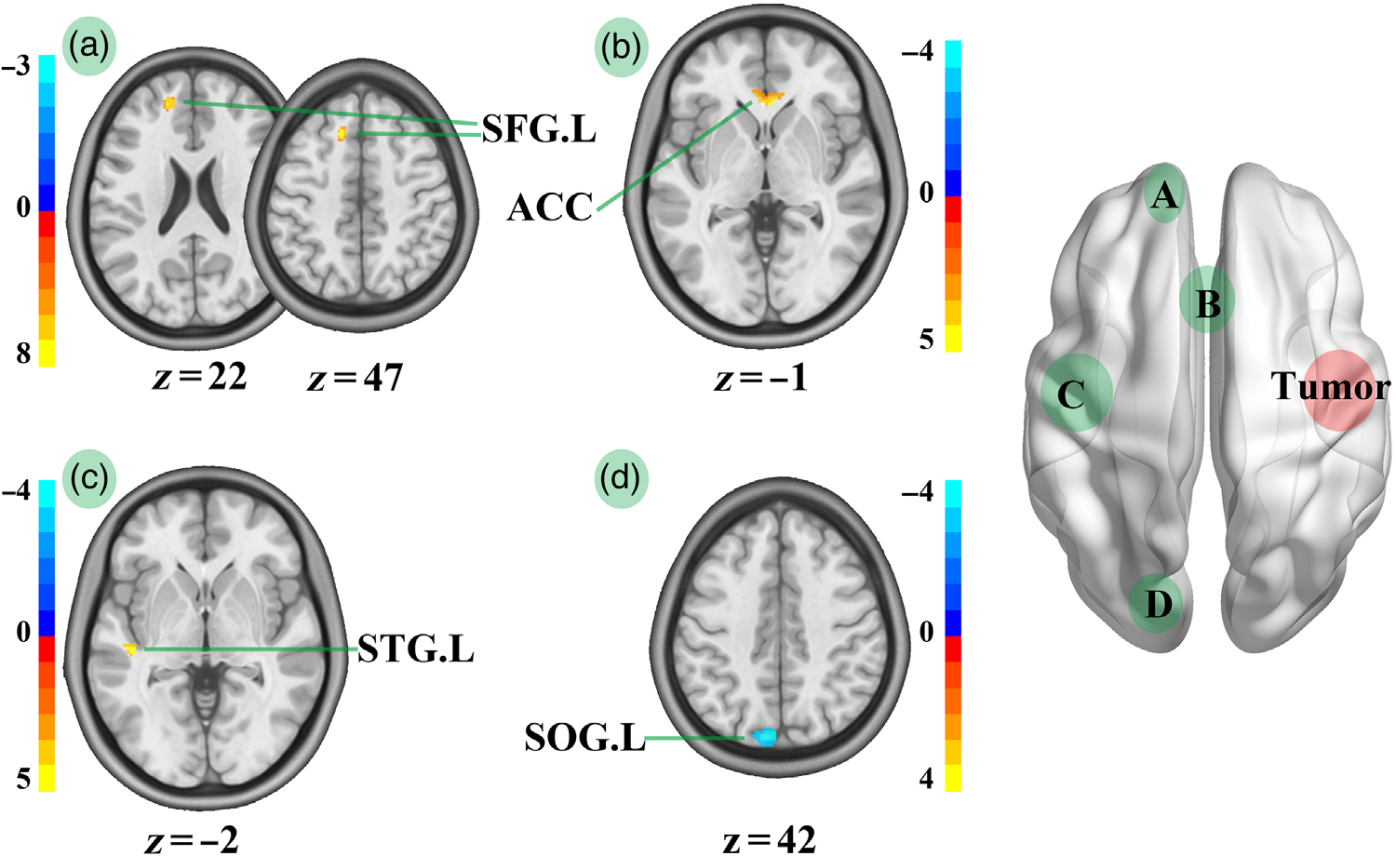
FA map comparison between HCs and patients with right insula glioma. STG.L, left superior temporal gyrus; SFG.L, left superior frontal gyrus; SOG.L, left superior occipital gyrus; RIGs, right insula gliomas; LFG, left front gyrus; LOG, left occipital gyrus; LTI, left temporal insula; ACC, anterior cingulate. We compared FA between HCs and patients with right temporal insula glioma in LFG (A), ACC (B), LTI (C), and LOG (D). Compared with HCs, RIGs group showed decreased FA in STG.L, ACC, and SFG.L, but increased in SOG.L.

We performed voxel‐wise statistical analysis of FA images to compare HCs and patients with insular glioma. Compared with HCs, patients with LIG showed decreased FA in the STG.R, right middle temporal gyrus, ACC, right superior frontal gyrus, and right superior occipital gyrus (Table 3; Figure 5). Furthermore, compared with HCs, patients with RIG showed decreased FA in the left superior temporal gyrus, ACC, and left superior frontal gyrus, but increased FA in SOG.L (Table 3; Figure 6).

**TABLE 3:** Peak locations of regions: Comparison of FA between healthy controls and patients with glioma

	Areas	Peak location (MNI coordinates)			Cluster size (voxels)	*T*
		*X*	*Y*	*Z*		
LIGs	RTI mask					
	STG.R	36	−14	6	1382	6.8745
	MTG.R	48	−48	8	206	4.3597
	MTG.R	42	−50	12	31	4.6948
	ACC mask					
	ACC	8	6	28	873	6.1177
	RFG mask					
	SFG.R	16	54	30	57	4.7638
	SFG.R	18	22	46	738	5.4195
	ROG mask					
	SOG.R	30	−74	18	340	4.5922
RIGs	LTI mask					
	STG.L	−46	−24	−2	41	5.0318
	ACC mask					
	ACC	−6	4	28	301	5.5242
	LFG mask					
	SFG.L	−18	48	22	42	4.4964
	SFG.L	−14	26	46	55	5.3422
	LOG mask					
	SOG.L	−8	−82	44	131	−4.0146

*Note*: Compared with HCs, LIGs group showed decreased FA in STG.R, MTG.R, ACC, SFG.R, and SOG.R. Compared with HCs, RIGs group showed decreased FA in STG.L, ACC, and SFG.L, but increased FA in SOG.L.

Abbreviations: ACC, anterior cingulate; FA, fractional anisotropy; LFG, left front gyrus; LIGs, left insula gliomas; LOG, left occipital gyrus; LTI, left temporal insula; MNI, Montreal Neurological Institute; MTG.R, right middle temporal gyrus; RFG, right front gyrus; RIGs, right insula gliomas; ROG, right occipital gyrus; RTI, right temporal insula; SFG.L, left superior frontal gyrus; SFG.R, right superior frontal gyrus; SOG.L, left superior occipital gyrus; SOG.R, right superior occipital gyrus; STG.L, left superior temporal gyrus; STG.R, right superior temporal gyrus.

## 4 DISCUSSION

In the present study, we investigated the alterations of the topological network and the changes in brain structure in patients with insular glioma. We found that patients showed topological network decline mainly in the insula, basal ganglia region, and ACC, which indicated that the tumor mainly disrupted the integrity of the topological network in the SN. We detected compensatory increases in GM volume mainly within the SN in glioma patients. Glioma patients also showed decreased GM volume in areas of the temporal cortices, suggesting decompensation of GM volume in patients with glioma. We mainly found FA decline in the SN, as well as the frontal lobe and occipital lobe, which suggested a breakdown of the integrity of WM. Taken together, the decrease of FA in patients with insular glioma may indicate loss of integrity within the WM, which in turn caused the destruction of the topological network of WM within the SN. Ultimately, this may have led to the decrease in network efficiency and the compensatory increase of GM volume in the SN to maintain normal brain function.

We explored the topological network alterations in patients with insular glioma through DTI based on the AAL template. Different from functional networks, which are constructed by time series correlation of the brain activity between voxels (Fingelkurts et al., 2005; Ghinda et al., 2018; Kelly & Castellanos, 2014), a topological network is constructed by fiber tracking using FA values or WM fibers that, in this study, was between the 90 brain regions identified by the AAL template (Hu, Qian, et al., 2020; Li et al., 2021). Hu, Qian, et al. (2020) found a significant reduction of network efficiency in the corticosubcortical circuits in depressed patients with Parkinson's disease, which indicated disrupted integrity in large‐scale brain systems. Furthermore, Li et al. (2021) found that the core symptoms in patients with autism spectrum disorder were correlated with the WM topological properties generated from a topological network. Other studies also showed that the structural network was significantly disrupted in brain disorders such as small vessel disease, depression, and epilepsy (Chen et al., 2021; Gou et al., 2018; Xie et al., 2017; Yu et al., 2019). Interestingly, another study found that functional and structural network properties were related to age (Shah et al., 2018). These studies have demonstrated characteristic changes in structural networks, which may be associated with heritable behavioral phenotypes (Bohlken et al., 2014). Furthermore, alterations of topological networks also exist in patients with glioma. For example, researchers found disconnection between hub nodes in patients with glioma presenting with intraoperative stimulation‐related epilepsy (Yang, Zhou, et al., 2021). Similarly, Zhou et al. (2023) found that nonepilepsy groups showed damaged topological networks with lower efficiency and longer path length compared with epilepsy groups in patients with glioma. Consistent with previous studies, we found disrupted topological network decline mainly in the insula, basal ganglia region, and ACC, which were mainly localized in the SN, in patients with insular glioma. There are multiple networks in the brain, and the DMN, ECN, and SN are recognized as three of the most important (Ji et al., 2019). The SN is a network that plays a crucial role in processing complex tasks in the human brain and in switching modes between the DMN and ECN. The main role of the SN is to detect, filter, and highlight stimulation, recruit a part of the relevant functional network, and detect and integrate emotional and sensory stimulation (Song et al., 2021). The constituent brain regions of the SN are AI and ACC (Chong et al., 2017; Ku et al., 2020). Therefore, regional tumors not only altered the regions adjacent to the lesion but also affected more distant brain regions. In the present study, insular glioma mainly caused network destruction in the SN and some regions related to the insula lobe. In summary, insular glioma mainly caused disruption of the WM topological network in the SN, which may have led to the decline in network efficiency notable within the patients.

FA has been widely used to characterize WM integrity in brain disorders (Li et al., 2015). Researchers found FA alterations in the parahippocampal cingulum in patients with Alzheimer's disease, suggesting that FA may help in the diagnosis of Alzheimer's disease (Dalboni da Rocha et al., 2020). FA is also altered in other diseases, such as spinal cord injury, meningioma, and major depressive disorder (Cesme et al., 2021; Hermesdorf et al., 2017; Vedantam et al., 2014). Meanwhile, alterations of FA may be related to brain cognitive function (Molina et al., 2018; Raghavan et al., 2020). Previous studies have also found that reduced integrity of specific WM fiber tracts may relate to age in several hubs (Bennett & Madden, 2014; Porcu et al., 2021; Wassenaar et al., 2019). Regarding patients with glioma, researchers found different patterns of FA alterations in isodehydrogenase mutated compared with wild‐type patients, which may be related to different degrees of malignancy of different gene mutations (Jutten et al., 2021). In the present study, FA was significantly decreased within the SN and front lobe, but patients with left and right lesions showed different trends of FA alterations in the occipital lobe. In fact, several studies have shown that the insular is functionally complex, and lesions in the insular may affect the abilities of sensorimotor, olfactory, social–emotional, cognitive, vocabulary, and learning functions and verbal memory (Diener et al., 2012; Kurth et al., 2010; Mandonnet, 2019; Uddin et al., 2017; Wu et al., 2011; Zarino et al., 2021). Tumors located in the SN not only affected FA within the network but also affected FA in the frontal lobe, which is relatively correlated with the SN. Different alterations of FA in SOG indicated that tumors not only destroyed WM integrity but also may induce compensatory increases in FA of WM in some brain regions to compensate for the damaged WM integrity. However, in general, insular gliomas were responsible for a general downward trend in FA, which may affect brain function.

Consistent with previous studies (Almairac et al., 2018; Hu, Hu, et al., 2020), patients with glioma mainly showed compensatory increases in GM volume in the SN and frontal lobe. However, decreased GM volume also was evident in some regions of the temporal insula and SOG.L, suggesting a pattern of GM volume decompensation in patients with glioma (Yuan et al., 2020). A variety of brain diseases could cause structural changes in the brain, reflected by alterations in GM volume (Li, Wen, et al., 2022; Oschwald et al., 2019; Takamiya et al., 2021). Burrowes et al. (2021) found GM volume was decreased in the right dorsal medial prefrontal cortex in episodic migraine patients, and untreated patients showed lower GM volume compared with treated, which reflected the importance of treatment options at baseline. Furthermore, changes in GM volume are also evident in diseases of other systems, such as diabetes (Liu et al., 2020), and GM volume may also change with age (Hirst et al., 2021; Long et al., 2020), which reflects the dynamic patterns of brain structure. In the present study, increased GM volume suggests compensation, while decreased GM volume suggests decompensation. In general, increases in GM volume mainly existed in the SN, reflecting compensation within this network in patients with insular glioma. The frontal lobe is the key region of the DMN and ECN, and the increase of GM volume in MFG.L indicated compensation of GM volume across these networks. Overall, the increase in GM volume in the SN and frontal regions may compensate for the structural alterations caused by a tumor.

## 5 LIMITATIONS

Despite our well‐designed experimental methods, there were still some limitations in our study. First, the population distribution was imbalanced among groups, with fewer HC than patients. However, we included age and sex as covariates in each step of the analysis to increase the credibility of our results. Next, since this was a retrospective study, the previous patients did not undergo corresponding cognitive function tests; thus, the physiological significance of changes in brain structure was not explored. However, we have been unifying the data, and we will explore the relationship between brain structure alterations and cognitive function in future studies. Finally, we included DTI images with different scan parameters in patients and HCs. Different parameters were used to optimize and improve the imaging protocol, which were independent of the purpose of the study. To avoid the influence of scan parameters in analysis, we conducted several steps in the preprocessing stage, including image normalization and registration to the standard space.

## 6 CONCLUSION

Through topological network analysis, we found that structural networks mainly deteriorated in tumor‐related nodes in patients with insular glioma, reflecting a trend of topological network destruction caused by the tumor. Using voxel analysis, we found that GM volume generally showed a compensatory increase within and across the network. Furthermore, FA decreased mainly in the SN, suggesting that glioma destroys the integrity of WM fibers. The insular serves complex functions, which are involved in sensorimotor, olfactory, social–emotional, cognitive, language, vocabulary, and learning functions and verbal memory. In summary, in patients with insular glioma, the decrease of FA indicated decreased integrity of WM tracts, which led to the destruction of the topological network within the SN and to the decrease of network efficiency and brain function. Finally, this can result in increased GM volume within or across the network to compensate for the decline of the network and function. This may provide some new insights into our understanding of functional compensation and show some auxiliary suggestions for protecting brain function in surgery in patients with insular glioma.

## AUTHOR CONTRIBUTIONS

Guanjie Hu, Junxia Zhang, and Yongping You contributed to the conception and design of the study, curated the data, wrote sections of the manuscript, performed statistical analyses and data interpretation, and wrote the first draft of the article. All authors contributed to manuscript revision and have read and approved the submitted version.

## CONFLICT OF INTEREST STATEMENT

The authors declare no conflicts of interest.

### PEER REVIEW

The peer review history for this article is available at https://publons.com/publon/10.1002/brb3.2969.

## Supporting information


*Supplementary materials S1. Image Preprocessing*

## Data Availability

All identified data used in the current manuscript can be provided by the authors per the readers’ requests. No data spreadsheet has been made publicly available due to patient confidentiality.
