# Supplementary material for: Structural alterations of the salience network in patients with insular glioma
Source: Brain Behav. 2023 Mar 28;13(5):e2969. doi: 10.1002/brb3.2969 (PMC10175985; doi:10.1002/brb3.2969)
Supplement: Supplementary file 1 — Supplementary materials S1. Image Preprocessing [file BRB3-13-e2969-s001.docx]

***Supplementary materials S1. Image Preprocessing***

The 3D T1-weighted images were preprocessed for Voxel-based morphometry (VBM) analyses using the Data Processing and Analysis of Brain Imaging (DPABI) [1] based on the Statistical Parametric Mapping (SPM) program, version 12 (SPM12; Statistical Parametric Mapping, http://www.fifil.ion.ucl.ac.uk/spm) implemented in MATLAB2022a (http://www.mathworks.com/products/matlab/) with the following preprocessing steps: first, we manually selected the anterior commissure as the origin (coordinate 0,0,0); next, each image was segmented into gray matter (GM), white matter (WM), and cerebrospinal fluid (CSF) using SPM8. The Diffeomorphic Anatomical Registration Through Exponentiated Lie Algebra (DARTEL) algorithm was then used to spatially normalize the segmented images [2]. Then, these images were spatially normalized, and the fully normalized images were resliced through trilinear interpolation to a final voxel size of 1.5 × 1.5 × 1.5 mm in MNI (Montreal Neurological Institute); an additional “modulation” step of multiplying each spatially normalized GM and WM image with its relative volume before and after normalization was also performed; finally, the resulting GM and WM images were smoothed using 8-mm full width at half-maximum (FWHM) Gaussian smoothing.

1. Yan, C.G., et al., *DPABI: Data Processing & Analysis for (Resting-State) Brain Imaging.* Neuroinformatics, 2016. **14**(3): p. 339-51.

2. Ashburner, J. and K.J. Friston, *Computing average shaped tissue probability templates.* Neuroimage, 2009. **45**(2): p. 333-41.
